# Morphometric and genetic evidence for four species of gentoo penguin

**DOI:** 10.1002/ece3.6973

**Published:** 2020-11-05

**Authors:** Joshua Tyler, Matthew T. Bonfitto, Gemma V. Clucas, Sushma Reddy, Jane L. Younger

**Affiliations:** ^1^ Department of Biology & Biochemistry Milner Centre for Evolution University of Bath Bath UK; ^2^ Department of Biology Loyola University Chicago Chicago IL USA; ^3^ Cornell Lab of Ornithology Cornell University Ithaca NY USA; ^4^ Cornell Atkinson Center for a Sustainable Future Cornell University Ithaca NY USA; ^5^ Bell Museum of Natural History Department of Fisheries, Wildlife, and Conservation Biology University of Minnesota St. Paul MN USA

**Keywords:** Antarctica, integrative taxonomy, new species, *Pygoscelis*, Southern Ocean

## Abstract

Gentoo penguins (*Pygoscelis papua*) are found across the Southern Ocean with a circumpolar distribution and notable genetic and morphological variation across their geographic range. Whether this geographic variation represents species‐level diversity has yet to be investigated in an integrative taxonomic framework. Here, we show that four distinct populations of gentoo penguins (Iles Kerguelen, Falkland Islands, South Georgia, and South Shetlands/Western Antarctic Peninsula) are genetically and morphologically distinct from one another. We present here a revised taxonomic treatment including formal nomenclatural changes. We suggest the designation of four species of gentoo penguin: *P. papua* in the Falkland Islands, *P. ellsworthi* in the South Shetland Islands/Western Antarctic Peninsula, *P. taeniata* in Iles Kerguelen, and a new gentoo species *P. poncetii,* described herein, in South Georgia. These findings of cryptic diversity add to many other such findings across the avian tree of life in recent years. Our results further highlight the importance of reassessing species boundaries as methodological advances are made, particularly for taxa of conservation concern. We recommend reassessment by the IUCN of each species, particularly *P. taeniata* and *P. poncetii*, which both show evidence of decline.

## Graphical abstract

Gentoo penguins (*Pygoscelis papua*) are found across the Southern Ocean with a circumpolar distribution and notable genetic and morphological variation across their geographic range. Here, we show that four distinct populations of gentoo penguins (Iles Kerguelen, Falkland Islands, South Georgia, and South Shetlands/Western Antarctic Peninsula) are genetically and morphologically distinct from one another. We suggest the designation of four species of gentoo penguin: *P. ellsworthi* in the South Shetland Islands/Western Antarctic Peninsula, *P. taeniata* in Iles Kerguelen, and *P. poncetii* in South Georgia with the Falkland Islands retaining *P. papua*.

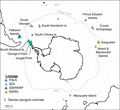

## 1 INTRODUCTION

A recent investigation into global species diversity of birds proposed that the number of species may be underestimated by as much as a factor of two when unrecognized species are accounted for (Barrowclough et al., 2016). This discrepancy exists in part due to the historical application of the Biological Species Concept (BSC) in ornithology. The BSC defines a species as a “group of actually or potentially interbreeding natural populations, which are reproductively isolated from other such groups” (Mayr, 1942). While generally applicable, the BSC is complicated in ornithology by the ability of birds to hybridize with deeply divergent relatives (Prager & Wilson, 1975). It is also often impossible to test for reproductive isolation in wildlife taxa that do not have overlapping ranges. As a result, the widespread application of the BSC led to an underestimation of avian species diversity. The Phylogenetic Species Concept (PSC), conceived by Cracraft (1983) and applied in Barrowclough et al. (2016), defines a species as “the smallest diagnosable cluster of individual organisms within which there is a parental pattern of ancestry and descent” or more simply as “a group of organisms that have a unique and shared evolutionary history (i.e., monophyletic).” This definition allows for species delimitation without the need to invoke reproductive isolation. Another factor leading to the recognition of greater avian species diversity is the advancement of species delimitation tools, including genomic sequencing and multivariate morphometrics. Unrecognized (or hidden) species are distinguishable using physical characters but were not previously recognized as full species due to either limitations in analytical methods or historical interpretations of the species concept. Cryptic species, on the other hand, refers to taxa that cannot be readily identified using physical characters, but can be discerned using genetic and/or ecological evidence (Hosner et al., 2018). Cryptic and hidden diversity in birds has been uncovered across the world in recent years by using the PSC in conjunction with integrative taxonomic approaches combining genomics and morphometrics, particularly in biodiversity hotspots such as the old‐world tropics and neotropics (Hosner et al., 2018; Pulido‐Santacruz et al., 2018; Singh et al., 2019; Younger et al., 2018, 2019). To manage conservation priorities in light of ongoing environmental change, it is vital to understand the true number of species that exist and their range limits, rather than relying on historic estimates.

**Figure 1:**
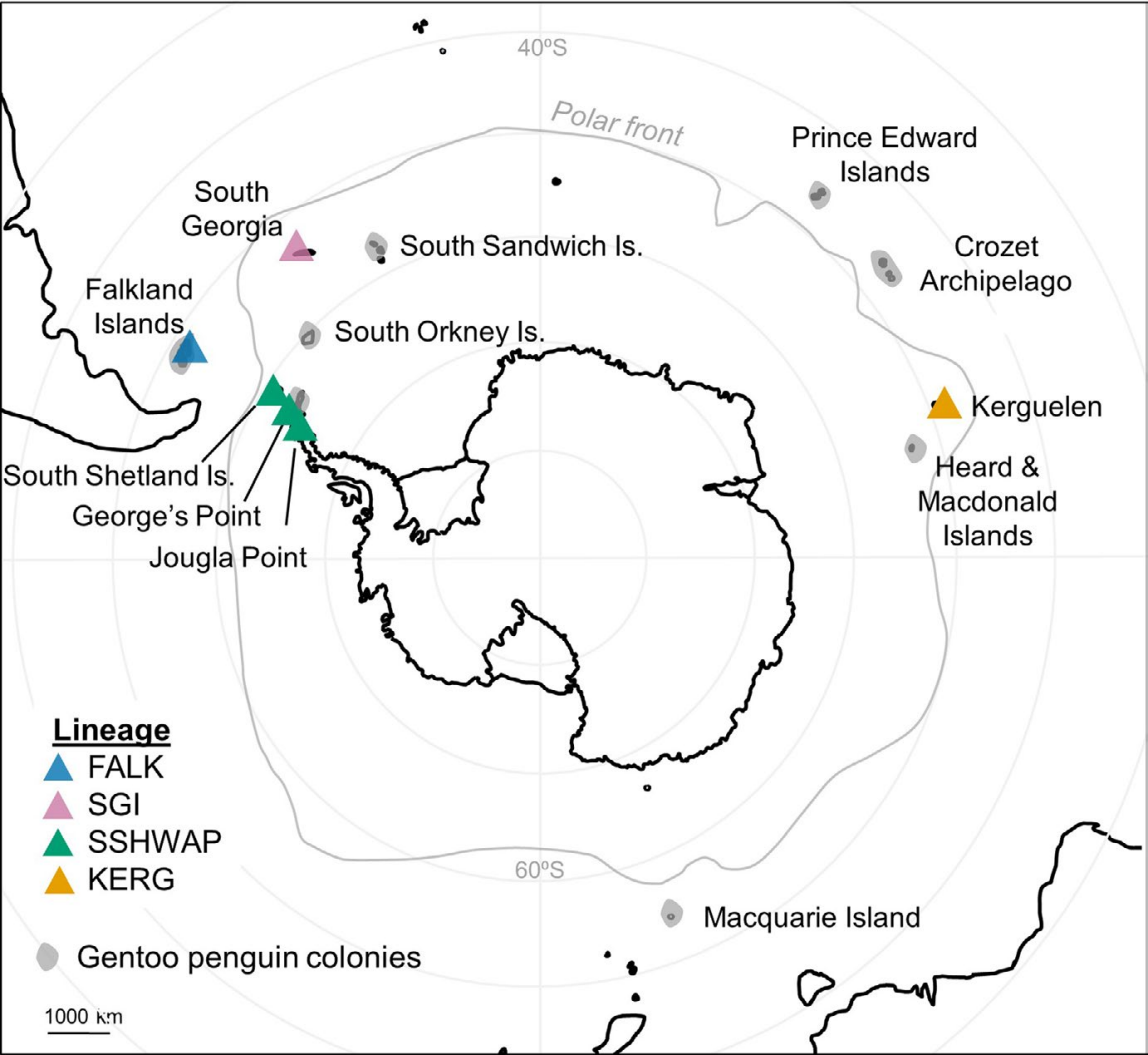
Geographic range of gentoo penguins. Gray zones show existing gentoo penguin colonies while colored triangles show populations included in this study. FALK, Falklands; SGI, South Georgia Island; SSHWAP, South Shetland Islands & Western Antarctic Peninsula; and KERG, Kerguelen

Given their large geographic range and already noted genetic and morphological differences (Clucas et al., 2018; Stonehouse, 1970), gentoo penguins could be strong candidates for harboring hidden species‐level biodiversity. First described by Forster (1781), the gentoo penguin (*Pygoscelis papua*) is the largest of the three *Pygoscelis* species and identifiable by its charismatic red‐toned bill, blackhead, and two contrasting white patches on the face. Gentoos have a circumpolar distribution spanning the Antarctic Convergence between 65°16’ S and 46°00’S, ranging from the Antarctic Peninsula to the Crozet Islands (Figure 1) (Forster, 1781; Lynch et al., 2012; Woehler, 1994). Given this geographic spread and the considerable heterogeneity in environmental conditions among extant populations, it is important to understand not only global trends in gentoo penguin numbers, but also how each of the individual populations is faring in the rapidly changing Antarctic climate. Individual populations may also provide evidence for how gentoo penguins adapt to specific environmental conditions which could be missed when generalizing over the polytypic species.

The global population size of gentoo penguins has increased sixfold over the past 40 years, despite a changing ecological landscape due to climate change (McMahon et al., 2019). Newly established colonies on the southern extent of the gentoo range seem to be growing due to the increasing breeding habitat brought about by receding sea ice (Juáres et al., 2019; Lynch et al., 2012). Established populations, however, show varying patterns of success, with populations at Port Lockroy, Kerguelen Island, and Macquarie Island seeing 1.4%, 2.3%, and 1.8% per annum decreases, respectively, based on multi‐decadal studies (Bingham, 1998; Dunn et al., 2018; Dunn et al., 2016; Juáres et al., 2019; Lescroël & Bost, 2006; Pascoe et al., 2020).

Several subspecies of gentoo penguin have been proposed over the past century; however, subspecies limits have differed depending on the author. These have been based on measurable phenotypic variation as there are no noted plumage differences among proposed taxa. The nominate subspecies, *Pygoscelis papua papua*, was part of the original species description by Forster (1781). Mathews (1927) described *P. p. taeniata*, which included populations on Marion, Crozet, Heard, Kerguelen, & the Falkland Islands. These subspecies were redefined by Peters and Paynter (1934) who designated the populations of Macquarie, Heard, Kerguelen, and Marion Islands as *taeniata* while gentoos from the Falklands, South Orkney, South Shetland, South Georgia, and the Western Antarctic Peninsula were assigned to *papua*. The next update to the taxonomy was by Murphy (1947), who designated the subspecies *P. p. ellsworthi* for the populations on the South Shetland Islands and Western Antarctic Peninsula. Stonehouse (1970) then investigated the subspecies boundaries, focusing on morphological variation. Stonehouse focused on the classic hypothesis that revolved around the influence of the Antarctic Polar Front and the extent of pack ice on geographic variation in gentoos, and thus split *P. papua* into a northern (*P. p. papua*) and southern subspecies (*P. p. ellsworthi*), found north and south of 60°S, respectively, while discounting Mathews' or Peters' claim for an eastern subspecies *P. papua taeniata* (Mathews, 1927; Murphy, 1947; Peters & Paynter, 1934; Stonehouse, 1970). The analysis used a univariate approach based on six measures (culmen length, foot length, flipper length, flipper area, dorsal plumage, and ventral plumage) and confirmed a north/south gentoo split in line with the Antarctic Polar Front hypothesis, with the South Georgia Island population belonging to the northern subspecies. Individuals measured from Kerguelen and Macquarie Islands were found to be statistically indistinguishable in this study and were different only slightly from those from South Georgia and the Falkland Islands, and therefore were also included in the nominate northern subspecies *P. p. papua* (Stonehouse, 1970). A recent study found support for a new clade in the sub‐Antarctic Indian Ocean based on morphological analyses but was not formally assigned to a new subspecies (de Dinechin et al., 2012) while investigations into geographic variation in gentoo vocalizations found no patterns connected with regions or subspecies (Lynch & Lynch, 2017).

Recent genetic analyses from across the penguin family have uncovered significant genetic divergence among populations across the Southern Ocean (Clucas et al., 2018; Frugone et al., 2019; Levy et al., 2016; Pertierra et al., 2020; Vianna et al., 2017). These studies, in combination with documented regional heterogeneity in population responses to climate change, highlight the importance of interrogating traditional ideas of subspecies limits within gentoo penguins (Levy et al., 2016; Vianna et al., 2017). Both Clucas et al. (2018) and Pertierra et al. (2020) suggested that cryptic species of gentoo penguins exist based on genetic methodology. Using an integrative taxonomic framework combining contemporary multivariate morphological analyses with previous genomics results, we aim to test whether the four genetic lineages of gentoo penguins described in Clucas et al., 2018 (Kerguelen, Falklands, South Georgia, and South Shetlands/Western Antarctic Peninsula) are morphologically distinct and therefore warrant recognition as distinct species under the Phylogenetic Species Concept. We then take the next step of formally describing distinct species so they will be included in assessment frameworks such as the IUCN Red List and conservation plans.

## 2 MATERIALS AND METHODS

### 2.1 Taxon sampling

Our geographic sampling within gentoo penguins (Figure 1) includes representatives from Kerguelen, the Falkland Islands, South Georgia, South Shetland Islands, and the West Antarctic Peninsula. This sampling spans the two currently recognized subspecies, namely the northern gentoo (the nominate subspecies, *Pygoscelis papua papua* (Forster, 1781)) distributed north of 60°S; and the southern gentoo (*Pygoscelis papua ellsworthi*), distributed on the Antarctic Peninsula and maritime Antarctic islands south of 60°S (Clements et al., 2019; Murphy, 1947; Stonehouse, 1970). Additionally, we include the putative Indian Ocean subspecies (de Dinechin et al., 2012), which is still classified as *P. p. papua* (Clements et al., 2019), and the South Georgia population, also classified as *P. p. papua*, but which multiple genetic studies show to be more closely related to *P. p. ellsworthi* (Clucas et al., 2014, 2018; Levy et al., 2016). Previous work has shown that gentoo penguin colonies on the South Shetlands and West Antarctic Peninsula are not reciprocally monophyletic in phylogenetic analyses (Clucas et al., 2018; Vianna et al., 2017); therefore, here, we group these populations into one unit for the purposes of this species delimitation study. There are therefore four putative species to be assessed: South Shetlands and the West Antarctic Peninsula (SSHWAP); Kerguelen (KERG); South Georgia (SGI); and the Falklands (FALK).

For genetic analyses, we used a published dataset (Clucas et al., 2018) of RAD‐Seq generated single nucleotide polymorphisms (SNPs). The dataset consists of 10,108 neutral SNPs for 69 gentoo penguins, with a median SNP coverage of 27X and a mean minor allele frequency (MAF) of 0.091 (*SD* 0.11). For morphometric comparisons, we measured all study skins of adult gentoo penguins from Kerguelen, the Falkland Islands, South Georgia, South Shetlands, and West Antarctic Peninsula available in the Natural History Museum (Tring, UK) and American Museum of Natural History (New York, USA) collections, totaling 39 individuals (Table S1). Birds with evidence of juvenile plumage were excluded.

### 2.2 Genetic variation

We used Genodive (Meirmans & Van Tienderen, 2004) to calculate the Weir and Cockerham unbiased weighted *F_ST_* estimator (Weir & Cockerham, 1984) between all pairs of populations, with significance calculated using 10,000 permutations of the data. Expected heterozygosity (*H_S_*) was also calculated for each population using Genodive. Principal components analysis (PCA) was used to visualize genetic variation among all individuals, using the *adegenet* package (Jombart, 2008; Jombart & Ahmed, 2011) in R. Allele frequencies were scaled and centered, and missing values replaced with the mean allele frequency using the *scaleGen* function. PCA was computed with the *dudi.pca* function from the *ade4* v1.7‐11 package.

We previously carried out maximum likelihood (ML) phylogenetic analysis and Bayes factor species delimitation for the gentoo penguin SNP dataset (Clucas et al., 2018). In brief, we used RAxML v8.2.7 (Stamatakis, 2014) to infer an ML phylogeny with a SNP ascertainment bias correction applied to the likelihood calculations. 20 independent ML tree inferences were carried out using the GTRGAMMA model and then the best scoring topology identified and annotated with bootstrap supports from 1,000 replicates. Coalescent‐based, Bayes factor species delimitation was carried out using the BFD* method (Leaché et al., 2014) as implemented within the SNAPP package (Bryant et al., 2012) of BEAST 2.4.3 (Bouckaert et al., 2014). The BFD* method estimates marginal likelihoods for competing species delimitation models using path sampling. We used four representative individuals for each lineage (KERG, FALK, SGI, and SSHWAP) and tested four models: (a) the current taxonomy (*P. p. papua* vs. *P. p. ellsworthi*); (b) the three‐taxa model suggested by mitochondrial DNA studies (Clucas et al., 2018; Vianna et al., 2017), wherein SGI and SSHWAP are grouped together; (c) the four‐taxa model (FALK, KERG, SSHWAP, and SGI); (d) a two‐taxa model with all colonies grouped except for Kerguelen, which is the most divergent according to our other analyses. For expanded details of these analyses, please refer to Clucas et al., 2018.

### 2.3 Morphological variation

To determine whether genetic lineages are morphologically distinct, one of us (JY) took nine linear measurements from each museum study skin, representing key morphological traits of both the crania and postcrania (Baldwin et al., 1931): culmen length (CL; taken along the medial line), bill width at the base (BWB), bill height at gonys angle (BH), bill width at gonys angle (BWG), flipper width (FW; shortest distance from anterior surface of flipper above the radiale to the posterior side of the flipper), radius length (RL), manus length (ML; indent at radiale/radius/ulna to distal wing tip), tarsus length (TML; anterior surface), and middle toe length (MTL; digit I11 excluding nail). Measurements were taken with Mitutoyo Digital Callipers to an accuracy of 0.01 mm. All measurements were repeated three times, checked for outliers (by confirming that all measurements were within one standard deviation), and then averaged. The summary statistics of these measurements for each of the four clades are given in Table S1. All measures were log‐transformed before the analyses. To identify traits that significantly differed between sexes, we carried out an analysis of variance (ANOVA) of sex within lineage for each trait (Table S2). Our testing found that only Flipper Width had a statistically significant difference between sexes (*p* = .024). This trait was therefore excluded from subsequent analyses to remove any potential bias introduced by uneven sampling of sexes.

Both univariate and multivariate analyses were used to investigate morphological differentiation between lineages. We carried out pairwise ANOVAs to determine whether any individual traits differed among lineages, and pairwise multivariate analysis of variance (MANOVA) on the combined trait dataset to assess overall morphological differentiation, using the *F* statistic for significance testing. These analyses were performed using base R (R Core Team, 2013). Principal components analysis (PCA) and linear discriminant analysis (LDA) were used as dimension‐reduction methods to aid with visualization and prediction, with lineage as a grouping variable using the *fviz_pca_biplot* function in factoextra and *lda* function in MASS in R (Kassambara & Mundt, 2017; R Core Team, 2013; Venables & Ripley, 2002). Confusion matrices and cross‐validation tests were constructed and performed using *predict* function in the MASS package in R (Venables & Ripley, 2002).

## 3 RESULTS

### 3.1 Genetic variation

**Figure 2:**
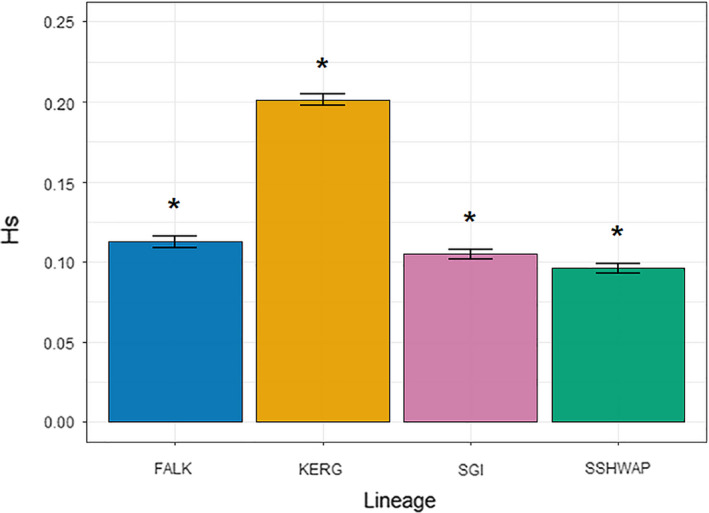
Heterozygosity. Genetic diversity (expected heterozygosity, Hs) of gentoo penguin lineage, with statistically significant differences indicated with asterisks

**Figure 3:**
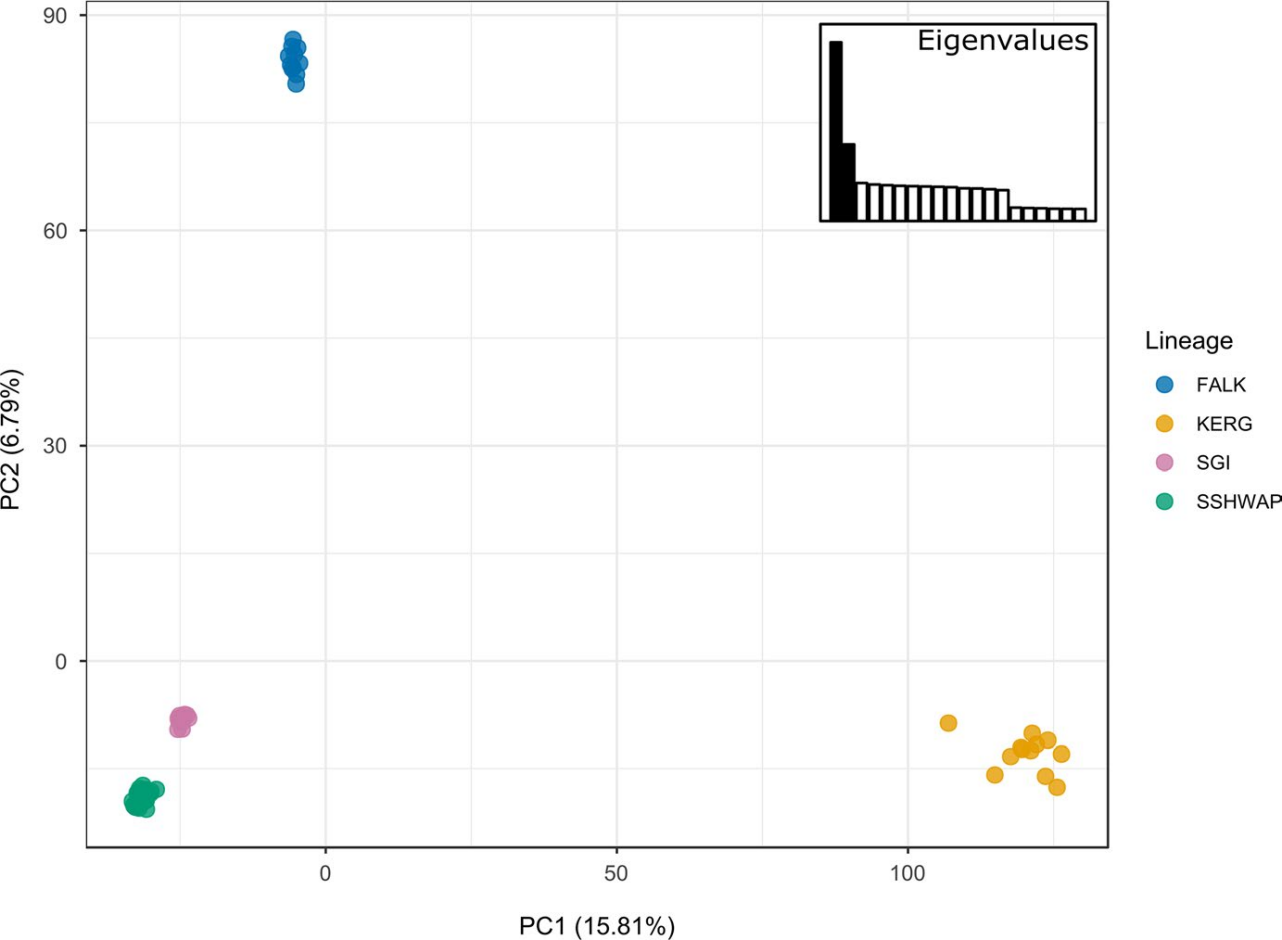
Principal Components Analysis based on genetic data. The amount of variance explained by each principal component (PC) is displayed on the inset bar graphs and on the axes, and the number of PCs retained is indicated in black

**Figure 4:**
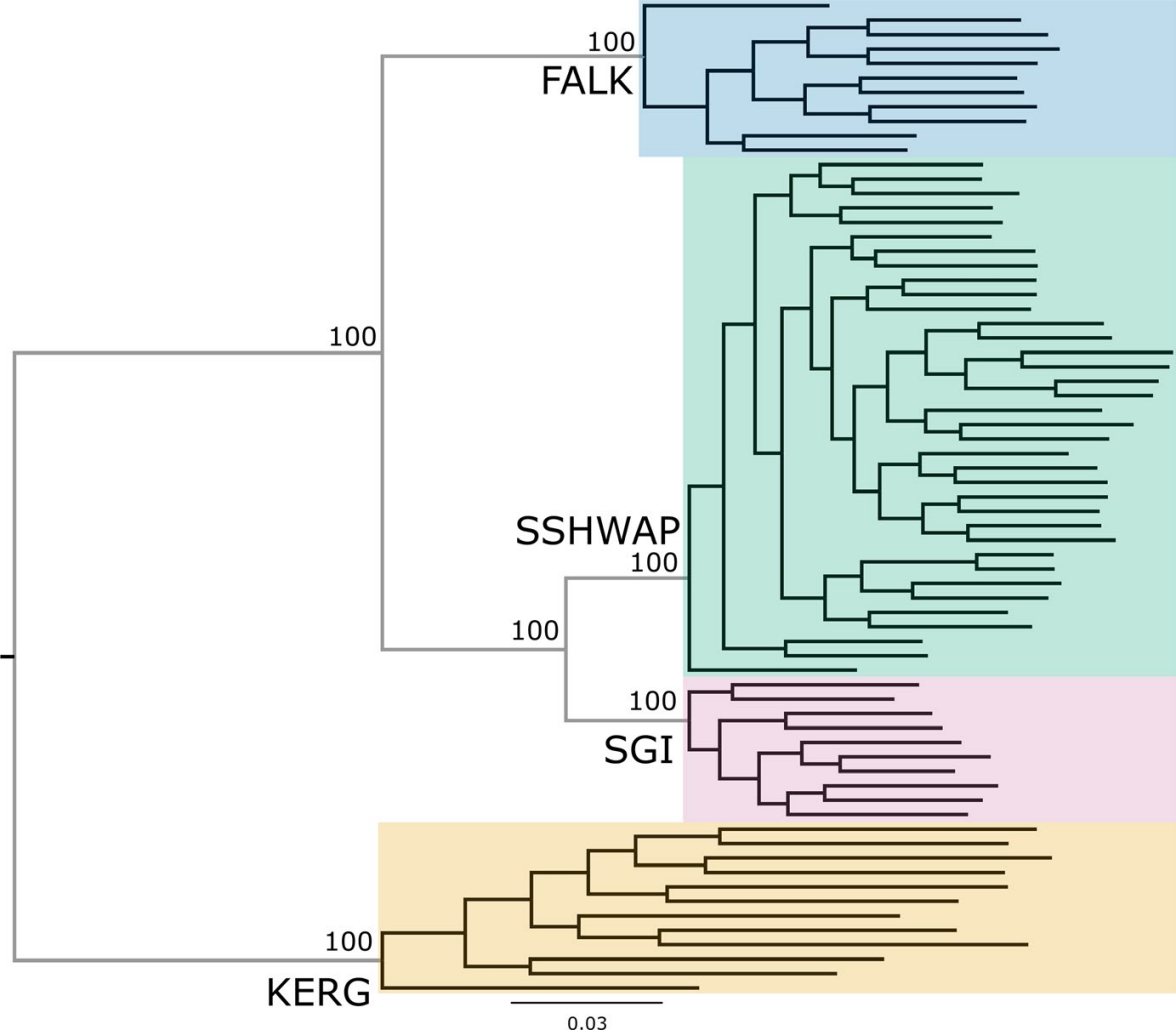
Best Scoring maximum likelihood phylogeny based on 10,108 neutral SNPs. Support values shown for branches that received >90% bootstrap support

All our genetic analyses show the four lineages (FALK, KERG, SGI, and SSHWAP) to be significantly genetically distinct. Pairwise *F_ST_* values among the four groups ranged from 0.130 to 0.341 and were all highly significant (*p* < .001, Table 1). Genetic diversity of the four lineages was all significantly different (Figure 2). Our PCA clearly differentiates the four lineages, with no evidence of overlap among the visible clusters (Figure 3). The ML phylogeny (Figure 4) resolved each lineage as 100% supported, with no well‐supported (>70%) splits within any of the four lineages. Our coalescent‐based species delimitation supported the four‐taxa model over all other models. The comparison of marginal likelihoods gave a Bayes factor of 17,595 for the four‐taxa model compared to the current taxonomy, and of 1,231 over the next most supported model (the three‐taxa model) (Table 2). Note that a Bayes factor of 10 is considered decisive (Kass & Raftery, 1995). The currently accepted taxonomy had the lowest marginal likelihood estimate.

**Table 1:** Pairwise FST values between all gentoo penguin populations

	FALK	KERG	SGI	SSHWAP
FALK	***	<0.001	<0.001	<0.001
KERG	0.26	***	<0.001	<0.001
SGI	0.247	0.265	***	<0.001
SSHWAP	0.281	0.341	0.13	***

Note

FST values are below the diagonal with *p* values above. No correction for multiple tests was performed as the range of the *p* values was too small.

**Table 2:** Path sampling results for four species delimitation models

Rank	Model	Number of taxa	MLE	BF
1	four nuclear clades	4	−83455.94	–
2	mitochondrial DNA hypothesis	3	−84071.77	1,231.67
3	Kerguelen versus. all others	2	−87197.45	7,483.02
4	current taxonomy	2	−92253.54	17,595.2

Note

All Bayes factor (BF) calculations are made against the most strongly supported model.

Abbreviation: MLE, Marginal likelihood estimate.

### 3.2 Morphological variation

**Figure 5:**
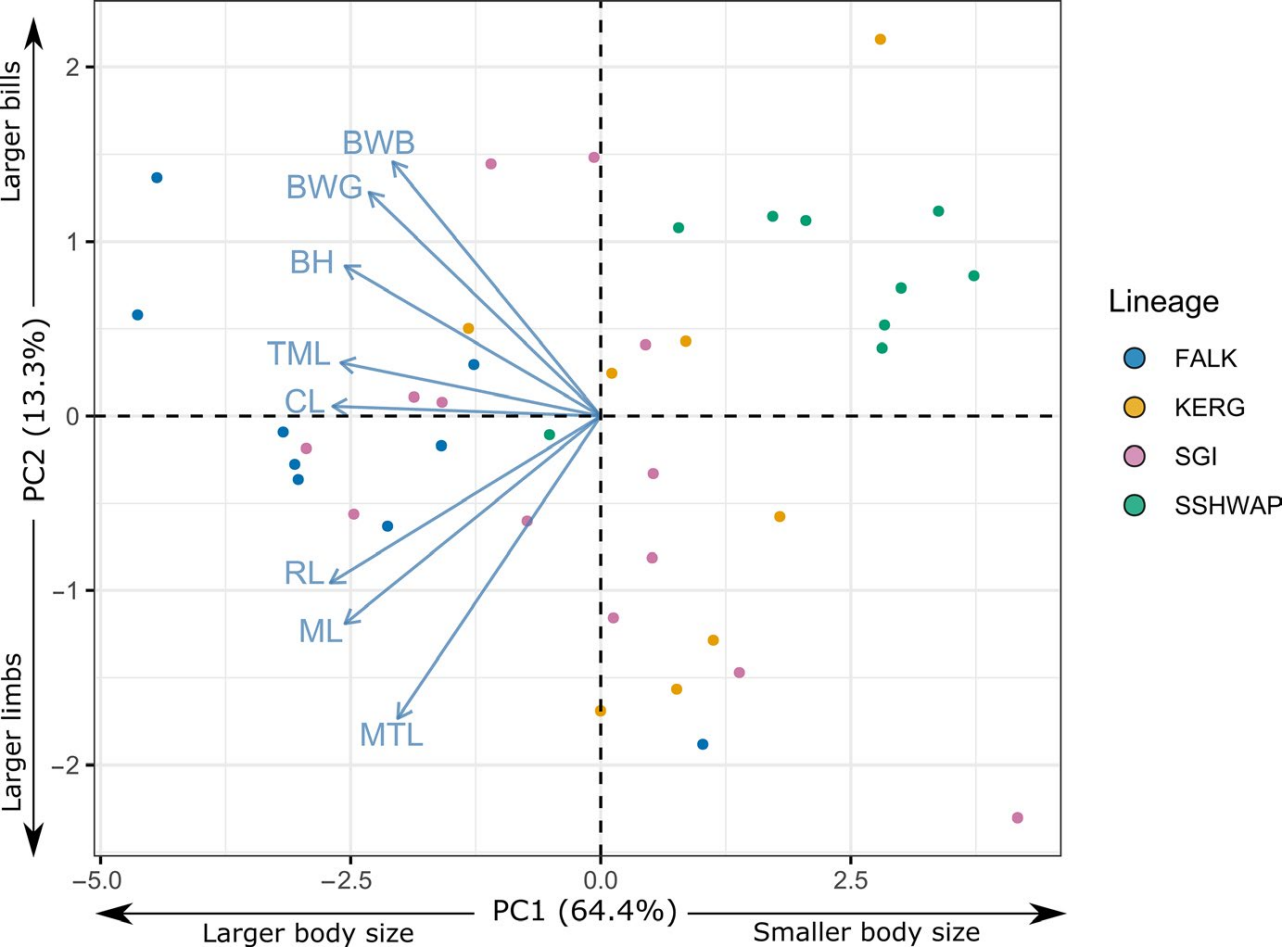
Principal Components Analysis of linear morphometrics. BH, bill height; BWB, bill width at base; BWG, bill width at gonys angle; CL, culmen length; ML, manus length; MTL, middle toe length; RL, radius length; TML, tarsus length

**Figure 6:**
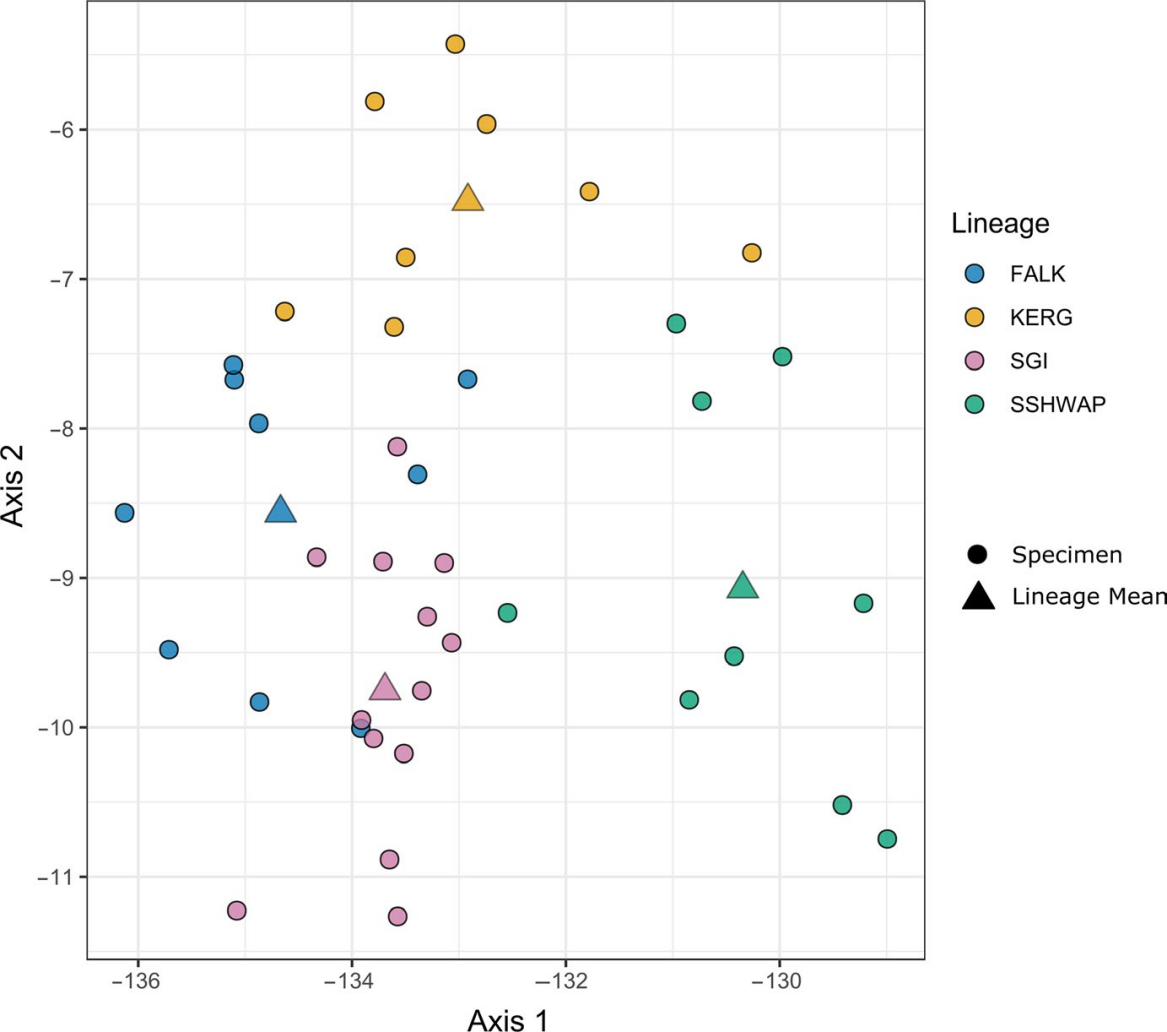
Linear Discriminant Analysis of linear morphometrics. Circles represent individual specimens with triangles showing the lineage mean

Our pairwise MANOVA tests determined that all genetically distinct populations are significantly morphologically distinct from each other overall (*p* < .05; Table 3). Our PC and LD analyses show some overlap in morphospace among the four lineages (Figures 5 and 6). In the PC analysis, PC1 explains 64.4% of the overall variation and is dominated by a size signal, with all traits increasing in size with increasingly negative PC1 scores. PC2 accounts for 13.3% of the variation and shows a split between cranial and postcranial measures, with all limb measures excluding tarsus length increasing with negative PC2 scores and all bill measures increasing with increasingly positive scores. On visual inspection of the LDA, the lineages are predominantly separate, with a small number of specimens occupying positions closer to other lineages. This is supported by the confusion matrices which found an error rate of 10.2% for the whole dataset and 35.9% with cross‐validation (Table S3).

**Table 3:** Pairwise MANOVA results between all gentoo penguin populations for linear traits

	FALK	KERG	SGI	SSHWAP
FALK	***	**0.0446**	**0.0328**	**0.0017**
KERG	3.5921	***	**0.0008**	**0.0381**
SGI	3.1375	7.9891	***	**0.0003**
SSHWAP	9.0501	3.8098	9.0188	***

Note

Approx. *F* values are below the diagonal with *p* values above. *p* values below .05 are indicated in bold.

Abbreviations: FALK, Falkland Islands; KERG, Kerguelen Island; SGI, South Georgia Island; SSHWAP, South Shetlands/Western Antarctic Peninsula.

Our pairwise ANOVA testing of traits showed that several individual traits enable discrimination of all four lineages (Table 4). The South Shetlands/West Antarctic Peninsula lineage is smaller than all other lineages and can be differentiated from South Georgia, the most closely related lineage in our genetic analyses, by its significantly smaller Culmen Length, Radius Length, Manus Length, Tarsus Length, and Middle Toe Length. The Falklands Islands birds are significantly larger than the other lineages for the majority of our measured traits. The Kerguelen and South Georgia lineages are intermediate in size and most similar to each other in our morphometric comparisons but can be differentiated by the significantly larger Manus Length and Middle Toe Length of the South Georgia lineage.

**Table 4:** ANOVA *p* values between all gentoo penguin populations for individual traits

Lineage		Trait							
A	B	CL	BWB	BH	BWG	RL	ML	TML	MTL
FALK	KERG	0.0818	**0.0186**	**0.0046**	**0.0259**	**0.0063**	**0.0032**	**0.0011**	**0.0088**
FALK	SGI	0.0894	0.1339	**0.0208**	0.2493	**0.0003**	**0.0292**	**0.0242**	0.1997
FALK	SSHWAP	**0.0001**	0.0528	**0.0031**	**0.0384**	**0.0000**	**0.0000**	**0.0001**	**0.0006**
KERG	SGI	0.9107	0.1126	0.9795	0.1377	0.6134	**0.0232**	0.1164	**0.0286**
KERG	SSHWAP	**0.0005**	0.3824	0.3615	0.4174	**0.0056**	**0.0020**	0.5032	0.6737
SGI	SSHWAP	**0.0045**	0.4127	0.4413	0.2631	**0.0039**	**0.0000**	**0.0233**	**0.0024**

Note

*p* values below .05 are indicated in bold. Culmen length (CL; taken along the medial line), bill width at the base (BWB), bill height at gonys angle (BH), bill width at gonys angle (BWG), flipper width (FW; shortest distance from anterior surface of flipper above the radiale to the posterior side of the flipper), radius length (RL), manus length (ML; indent at radiale/radius/ulna to distal wing tip), tarsus length (TML; anterior surface), and middle toe length (MTL; digit I11 excluding nail).

Abbreviations: FALK, Falkland Islands; KERG, Kerguelen Island; SGI, South Georgia Island; SSHWAP, South Shetlands/Western Antarctic Peninsula.

## 4 DISCUSSION

Our integrative taxonomic approach has revealed four deeply divergent lineages within gentoo penguins. These lineages are associated with different regions in the Southern Ocean, formed reciprocally monophyletic clades and genetic clusters in all our analyses, and are morphologically distinct. The clusters found here differ slightly from those found in other recent studies. Pertierra et al. (2020) studied individuals from three of the four lineages presented here alongside further island populations. Although they lacked samples from South Georgia and the South Sandwich Islands, they proposed a single Antarctic clade, grouping the Antarctic Peninsula, South Georgia, South Orkney Islands, and South Sandwich Islands. This study finds that there is cryptic diversity within this clade, with the Antarctic Peninsula and South Georgia being both morphologically and genetically distinct from each other. Interestingly, when studying the usefulness of cranial versus postcranial traits in separating lineages, postcranial ANOVA tests produce far more significant results (19 out of 24, *p* < .05) and are able to separate all pairwise lineages in comparison with the cranial trait tests (9 out of 24) which all failed to significantly separate the Kerguelen and South Georgia lineages. This strengthens the argument of not limiting analyses to only beak measures and instead including traits from the whole body. Given the evidence presented here, and the need to account for all species‐level diversity in conservation planning, we recommend that the *Pygoscelis* genus be revised to include four species of gentoo penguin.

There are currently two recognized subspecies of gentoo penguin: *P. papua papua* and *P. papua ellsworthi*, representing the classic north/south split within gentoos (Stonehouse, 1970). Other subspecies of *P. papua* have been previously proposed, including *P. papua taeniata*, which has included various combinations of island populations since its inception in 1927 (Mathews, 1927; Peters & Paynter, 1934). The Falkland Islands lineage will retain the name *P. papua*, given that *P. papua* was originally described from the Falkland Islands (Forster, 1781). The South Shetland Islands and Western Antarctic Peninsula lineage are currently considered as subspecies *P. p. ellsworthi;* therefore, we propose that this lineage be elevated to a full species named *P. ellsworthi*. Based on previous genetic work, we conclude that the South Orkney Islands gentoos also belong to the *P. ellsworthi* lineage (Pertierra et al., 2020). The Kerguelen lineage was previously described as the subspecies *P. p. taeniata* (Mathews, 1927; Peters & Paynter, 1934), which fell out of usage in the 1970s (Stonehouse, 1970), following which the Kerguelen gentoos have been classified as *P. p. papua*. We suggest the Kerguelen lineage now be designated as *P. taeniata* accordingly. We note that Mathews (1927) and Peters and Paynter (1934) grouped Macquarie Island, Heard Island, and Marion Island gentoos in *P. p. taeniata* with those from Kerguelen based on morphology. A genome‐wide SNP study by Pertierra et al. (2020) showed that gentoos from Crozet and Marion Islands are effectively a single lineage, and Kerguelen Island gentoos are a distinct sister lineage. Analysis of Macquarie Islands gentoos is limited to mitochondrial DNA, but shows this lineage to be sister to Crozet/Marion gentoos (Pertierra et al., 2020). At this stage, we suggest that Crozet, Macquarie, and Marion Island gentoos are better considered as *P. taeniata* along with Kerguelen gentoos, rather than their current designation as *P. papua*, given the available evidence. However, this is subject to change pending detailed investigation with integrative taxonomic methods. Individuals from the South Orkneys, South Sandwich Islands, Price Edward Islands and Heard & Macdonald Islands should be assigned once further morphological and genome‐wide studies are conducted given their geographical proximity to multiple lineages. The South Georgia lineage is currently classified as *P. p. papua,* and to our knowledge, there have been no previous subspecies or species suggested for South Georgian gentoos. We therefore describe this for the first time as.

### 4.1 *Pygoscelis poncetii* sp. nov

*Common Name*. South Georgia gentoo penguin.

*Zoobank Registry:* urn:lsid:zoobank.org:pub:0DADF56F‐ADD6‐4A4C‐A1DF‐C18187700EF2.

*Holotype*. American Natural History Museum (AMNH) 132462. Adult male collected by Robert C. Murphy at South Georgia, South Atlantic Ocean on 11th March 1913. The specimen was prepared as a museum flat skin and was used in the morphological analysis.

*Paratypes*. Specimens used in the morphological analyses**. AMNH 132463, AMNH 132464, AMNH 132465**: Adult males collected by Robert C. Murphy at South Georgia, South Atlantic Ocean on 11th March 1913**. AMNH 269638**: Adult female collected by Robert C. Murphy at Possession Bay, South Georgia, South Atlantic Ocean on 12th March 1913. **AMNH 435821, AMNH 435822, AMNH 435823**: Adults collected by Robert C. Murphy at Possession Bay, South Georgia, South Atlantic Ocean on 13th March 1913. **AMNH 525826, AMNH 525827**: Collected on South Georgia Island. **Tring 1914_3_8_6, Tring 1914_3_8_7, Tring 1914_3_8_8**: Adult males & female collected by P. Stammwitz at King Edward Point, South Georgia, South Atlantic Ocean in November 1913.

*Etymology. Pygoscelis poncetii* is named after Sally Poncet, whose body of work has significantly influenced the field of polar biology, particularly in relation to South Georgia.

*Diagnosis*. Morphologically, *P. poncetii* can be differentiated from all other species of gentoo by its manus length (mean length = 130.49 mm), being significantly smaller than *P. papua* (mean length = 135.08 mm, *p* = .0292) and significantly larger than both *P. ellsworthi* and *P. taeniata* (mean lengths = 115.38 mm and 125.27 mm, *p* < .0001 and *p* = .0232, respectively). Radius length differentiates *P. poncetii* (mean length = 52.68 mm) from the larger *P. papua* (mean length = 58.11 mm, *p* = .0003) and smaller *P. ellsworthi* (mean length = 48.90 mm, *p* = .0039). Genetic comparative techniques (Pairwise *F_ST_*, heterozygosity, clustering methods) found significant differences among all four species of gentoo penguin with the maximum likelihood phylogeny resolving each species as 100% supported. There are no discernible differences in plumage patterns among the four species.

*Description of holotype*. Blackhead with white band over the crown from eyebrow to eyebrow. Back dark blue gray with white on the ventral side between breasts and vent. Flippers dark blue gray edged with white. Black‐tipped orange bill. Orange/pink feet.

*Measurements of the holotype*. Culmen length: 59.85 mm, bill width at base: 18.27 mm, bill height at gonys angle: 17.07 mm, bill width at gonys angle: 9.88 mm, flipper width: 54.53 mm, radius length: 55.48 mm, manus length: 139.50 mm, tarsus length: 36.75 mm, middle toe length: 77.24 mm.

*Description of paratypes*. No discernible variation in coloration was found in the proposed species, with all matching the description given for the holotype. Summarized as follows: Blackhead with white band over the crown from eyebrow to eyebrow. Back dark blue gray with white on the ventral side between breasts and vent. Flippers dark blue gray edged with white. Black tipped orange bill. Orange/pink feet.

*Comparisons*. The principal components analysis shows that size is the key delimiter among all species, with *P. ellsworthi* representing the smallest gentoo followed by *P. taeniata*, *P. poncetii,* and *P. papua*. The separation of all species is supported by the significant pairwise MANOVAs across the full morphological dataset (Table 3). The individual pairwise ANOVAs of the univariate measures show that the new *P. poncetii* can be distinguished from all other species by its manus length (mean = 130.49 mm, range = 124.79–139.50 mm), with *P. papua* exhibiting a larger size (mean = 135.08 mm, range = 124.47–144.46 mm, *p* = .0292) while *P. ellsworthi* (mean = 115.38 mm, range = 110.07–126.72 mm) and *P. taeniata* (mean = 125.27 mm, range = 114.15–133.40 mm) are significantly smaller (*p* < .0001, and *p* = .0232, respectively). These morphological results show broad agreement with the univariate testing performed by Stonehouse (1970) but are now confirmed with modern multivariate methods.

In addition to morphology and genetics, there are notable ecological differences among the lineages. These include breeding habitat, which splits the flat beach and tussock grass nests of South Georgia and the Falkland Islands (Croxall & Prince, 1980; Reilly & Kerle, 1981) from the low‐lying gravel beaches and dry moraines of the South Shetlands and West Antarctic Peninsula (Jablonski, 1984; Volkman & Trivelpiece, 1981). Lineages also differ in diet, particularly the proportions of crustaceans, fish, and squid consumed (Ratcliffe & Trathan, 2012). It has been observed that there is a trend of decreasing dietary variability and increasing krill consumption at higher latitudes (Bost & Jouventin, 1990). Importantly, several recent studies of gentoo penguin population sizes have reported very different trends, reinforcing the need to understand the risks to specific populations. Increases of 3.5% and 3.1% per annum have been recorded on the South Orkney Islands (*P. ellsworthi*) and South Shetland Islands (*P. ellsworthi*), respectively, while there has been a marked decrease of 1.4% and 2.3% per annum at Port Lockroy (a colony within *P. ellsworthi*) and across Kerguelen (*P. taeniata*), respectively, (Bingham, 1998; Dunn et al., 2018; Dunn et al., 2016; Juáres et al., 2019; Lescroël & Bost, 2006).

Gentoo penguins are currently listed as “Least Concern” on the IUCN Red List, with their last assessment in 2018 (BirdLife International, 2018). In order to be listed as Vulnerable, a species must exhibit one or more risk criteria, for example, population size reduction greater than 30%, a limited geographic range, small population size, or evidence of likely extinction in the next 100 years (IUCN, 2015). Here, we have shown that *P. papua* should be considered as at least four distinct species. While *P. papua* in the Falkland Islands and *P. ellsworthi* appear to be stable or increasing, (Baylis et al., 2011; Crofts & Stanworth, 2016; Dunn et al., 2016; Juáres et al., 2019), *P. taeniata* experienced a 30% reduction in numbers between 1987 and 2004 (Lescroël & Bost, 2006). *Pygoscelis poncetii* may also be declining at South Georgia (Woehler et al., 2001). These two species should therefore be high priority for reassessment by the IUCN.

## 5 CONCLUDING REMARKS

In this paper, we highlight hidden biodiversity within the species *P. papua* using genetic and morphometric methods, in keeping with recent assessments of hidden species diversity in birds. Our results clearly support the division of gentoo penguins into at least four species. We name a new species of gentoo, *P. poncetii*, and recommend elevation of three subspecies to species level (*P. taeniata, P. papua,* and *P. ellsworthi*). Our results show the importance of reassessing species boundaries as methodological advances are made. These findings have implications for the threat status of these species, and we urge that this diversity is considered in conservation planning for the Southern Ocean.

## CONFLICT OF INTEREST

None declared.

## AUTHOR CONTRIBUTIONS

**Josh Tyler:** Data curation (equal); formal analysis (equal); investigation (equal); visualization (equal); writing–original draft (lead). **Matthew T. Bonfitto:** Data curation (supporting); formal analysis (supporting). **Gemma Clucas:** Conceptualization (supporting); data curation (equal); formal analysis (equal); investigation (equal). **Sushma Reddy:** Conceptualization (supporting); data curation (equal); investigation (supporting). **Jane Younger:** Conceptualization (lead); data curation (equal); formal analysis (equal); investigation (equal); project administration (lead); supervision (lead); writing–original draft (supporting).

## Supporting information

Table S1

Table S2

Table S3

## Data Availability

Gentoo morphological data are provided in Supplementary Table A alongside accession numbers and location. Additional material is available from the Dryad Digital. Repository: https://doi.org/10.5061/dryad.bs30388 for gentoo SNP datasets (Adapted from Clucas et al. (2018) https://doi.org/10.1111/mec.14896).
